# Determination of Mercury Exposure among Dental Health Workers in Nakhon Si Thammarat Province, Thailand

**DOI:** 10.1155/2014/401012

**Published:** 2014-10-01

**Authors:** Somsiri Decharat, Piriyaluk Phethuayluk, Supandee Maneelok, Phayong Thepaksorn

**Affiliations:** ^1^Department of Industrial Hygiene and Health Science, Faculty of Health and Sports Science, Thaksin University, 222 Moo 2 Papayom District, Phatthalung 93110, Thailand; ^2^Department of Public Health, Faculty of Health and Sports Science, Thaksin University, Phatthalung 93110, Thailand; ^3^Sirindhorn College of Public Health, Trang 92110, Thailand

## Abstract

*Objectives*. The main objective of this study was to assess the mercury exposure levels in dental health workers that work in dental clinics. The study evaluated the airborne and urinary mercury levels, the type of work done in the clinic, and the effect of mercury exposure on health of dental health workers. *Material and Methods*. A case-control study was conducted with 124 exposed and 124 matched nonexposed subjects. Personal and area samplings were conducted to quantify mercury concentrations by solid sorbent tube. Urine samples were collected to determine mercury levels by cold-vapor atomic absorption spectrometer mercury analyzer. *Results and Discussion*. 17.6% (*n* = 32/182) of the air samples were higher than the occupational exposure limit (OEL). A multiple regression model was constructed. Significant predictors of urinary mercury levels included dietary consumption (fish or seafood), duration of work (yrs), work position, personal protection equipment used (PPE), and personal hygiene behaviors. Significant correlations were observed between mercury levels in urine and mercury in storage areas (*r* = 0.499, P < 0.05) and between mercury levels in urine and airborne mercury in personal samplings (*r* = 0.878, P < 0.001). *Conclusion*. Improvements in working conditions, occupational health training, and PPE use are recommended to reduce mercury exposure.

## 1. Introduction

Exposure to mercury by dental health workers is associated with amalgam restorations in dental practices. Dental amalgam is a mixture of metals, consisting of liquid mercury and a powdered alloy composed of silver, tin, and copper. Approximately 50% of dental amalgam is elemental mercury by weight. During the amalgam preparation and tooth restoration process, the mercury vapor is emitted into the air [1, 2]. Studies have demonstrated that mercury exposure has effects on kidney function [3, 4] and on the central nervous system. Mercury also has been implicated in adverse effects on lung function, increased heart and blood pressure [5–9], and leukocytosis and neutrophilia [9].

Early symptoms can be unspecific and present as tiredness, loss of appetite, irritability, anxiety, agitation, and depression. Later symptoms can develop as memory loss, difficult sleep patterns, and personality changes [10]. The Occupational Safety and Health Administration (OSHA) [11] established the permissible exposure limit (PEL), the only legally enforceable federal U.S. standard, as a ceiling (i.e., level not to be exceeded) value of 100 *μ*g/m^3^ (actual standard is 1 mg/10 m^3^). NIOSH set a recommended exposure limit (REL) of 50 *μ*g/m^3^ as a 10-hour, time-weighted average. The American Conference of Governmental and Industrial Hygienists (ACGIH) recommended the most recent occupational exposure standard as the threshold limit value-time weighted average (TLV-TWA) of 25 *μ*g/m^3^ [12, 13]. The selection of biological samples to assess human exposure depends on the mercury compounds, exposure pattern, and time of sampling the exposure. Exposure to elemental mercury is well represented by the presence of mercury in urine. Urinary mercury is an indicator of average exposure during the past month rather than exposure at the time of urine collection. ACGIH recommended a biological exposure index (BEI) of total mercury in urine >20 *μ*g/g creatinine [14].

The main objectives of this study were to determine airborne mercury levels in dental clinics and the mercury concentrations in urine samples of dental health workers. In addition, we aimed assessing any associations between mercury levels in urine and airborne mercury concentrations in dental clinic, as well as with descriptive factors, such as demographics, job positions, working environments, and behavioral hygiene.

## 2. Material and Methods

### 2.1. Subjects

The study population consisted of dental health workers (16 dentists, 70 dental hygienists, and 38 dental assistants) who worked at 17 community hospitals in Nakhon Si Thammarat Province in the South of Thailand between May and September 2013.

124 exposed subjects were recruited. 30 exposed subjects were male and 94 exposed subjects were female. Control subjects, matched to exposed subjects by gender, were recruited from the workers who worked at the same community hospitals but had not had occupational contact with mercury. The inclusion criteria of the exposed group were dental health workers aged between 20 and 60 years who had experienced and contacted mercury on their daily routine work, for at least one year. They agreed to participate in the study and provided written informed consent.

### 2.2. Urine Collections

The 248 subjects (124 exposed and 124 unexposed) were interviewed using structured questionnaire interviews. Spot urine samples (30 mL) were collected that extended from the time the subjects went to bed through the first urination of the morning. The urine samples were kept in polypropylene sampling vessels and stored at −20°C prior to analysis.

### 2.3. Questionnaire

In the questionnaire interviews, detailed descriptive information was collected, including personal descriptive characteristics, dietary habit, occupational life styles, working positions, working environment, and personal hygiene. Direct observations were also made and recorded to confirm the questionnaire interviews. At the end of shifts, the subjects were also interviewed.

### 2.4. Area Air Mercury Vapor Samplings and Personal Air Sampling

Area air samples were taken at 3 areas within the dental healthcare office including the area around the base of the chair, the area around the storage area for waste amalgam, and the work surface where the preparation of amalgam usually taken place. Personal air samples were collected in the exposed subject's breathing zone. All samples were collected, with personal air samplers, for analysis of mercury concentrations by solid sorbent tube (hopcalite in single section, 200 mg, SKC Inc., PA, USA) (Gilian, Gilair-5RP Air Sampler). Before use, each air sampler was calibrated to obtain a flow rate of 0.2 L/min for a recommended sampling time of 8-hour time-weighted average. Samplers were attached to the pumps with flexible tubing and air was collected. Samples were capped and packed securely for shipment. The concentrations of mercury vapor were analyzed using cold-vapor atomic absorption spectrophotometer (CVAAS) (NOISH method 6009) [9].

## 3. Laboratory Analysis

### 3.1. Determination of Creatinine in Urine Samples

Creatinine in urine was measured using a test kit based on the Jaffé reaction. (Merckotest number 3385; Merc, Darmstadt, Germany).

### 3.2. Quantification of Mercury in Air Samples

Quantitatively transferred the hopcalite sorbent and the front glass wool plug from each sampler tube into a 100 mL volumetric flask. 2.5 mL of concentrated HNO_3_ and 2.5 mL of concentrated HCl were added, mixed, and allowed standing for a further 1 hour or until the black sorbent was dissolved. The solution's color changed to dark brown, which was carefully diluted to 50 mL with deionized water. This process was maintained until the blue-green color was sustained; then a further 2.5% w/w HNO_3_ and 2.5% HCl were added and mixed. Mercury in air samples was analyzed by CETAC M6000A cold-vapor atomic absorption spectrometer (CVAAS) mercury analyzer. This method of mercury in air samples (hopcalite in single section, 200 mg) determination was modified from NIOSH 6009 [15].

### 3.3. Validation of Mercury in Air Samples Analyses

CETAC M6000A cold-vapor atomic absorption spectrometer (CVAAS) was used for cold vapor analysis. Instrumental parameters were a slit width of 0.5 rim, wavelength of 253.7 nm, photo multiplier voltage of 4 mA, no background correction, and a delay time before reading of 55 or 70 sec. The delay time was reduced to 55 sec to reduce digest volume used in analysis. The limit of quantization (LOQ) corresponded to 0.01 absorbance units, which was produced by solutions containing 1.0 *µ*g Hg/L. Thus, the LOQ was established as 0.06 *µ*g Hg/g sample. The four-point calibration curve ranged from 1.0 to 40.0 *µ*g/L. The calibration curves were performed before each batch of analyses, and intermittent standards were analyzed every 10 samples. These measures assured instrumental accuracy and precision within and between days. The limit of detection (LOD) was 0.5 *µ*g/L. Triplicate readings were taken for each sample and averaged. Technique accuracy was well within the acceptable recovery range of 95% to 105%, and the precision was also within acceptable limits of 5% RSD.

### 3.4. Quantification of Urine Mercury Levels

Two milliliters of each urine sample was mixed with 0.1 mL of 35% w/w nitric acid, 0.2 mL of 50% w/w sulfuric acid, and 0.5 mL of 5% w/v potassium permanganate; then microwave digestion was carried out at an elevated temperature for 15 minutes. The sample solution was allowed standing at room temperature. If the solution's color changed from purple to brown, then a further 0.5 mL of permanganate solution was added, mixed, and allowed standing for a further 8 hours. This process of adding successive aliquots of permanganate solution and allowing the reaction to proceed was maintained until the purple color was sustained. With increasing masses of dissolved organic materials, increasing volumes of permanganate solution are required. After the permanganate reaction was completed, 0.4 mL of 2.5% (w/v) potassium persulfate was added and mixed; then it was placed in an incubator at 95°C for at least 2 hours before cooling down to room temperature. Next, 0.5 mL of 5% (w/v) hydroxylamine hydrochloride and 1 mL of 10% SnCl_2_ solution were added with an accessory dispenser. The total volume was made up to 10.0 mL with reagent water and mixed well prior to determination. This method of urinary mercury determination was modified from Ham, 1997 [16].

### 3.5. Validation of Mercury in Urine Analyses

Urine mercury was analyzed by CETAC M6000A cold-vapor atomic absorption spectrometer (CVAAS) mercury analyzer. Field blank samples and laboratory blank samples were used in all of the analyses as a quality control. Determination of urinary mercury levels was calibrated by preparing a series of standard additions containing 0, 10, 20, and 40 *µ*g/dL. The correlation coefficient (*r*) between the mercury concentrations in the authentic mercury solution and absorption was 0.9998. The limit of quantization (LOQ) corresponded to 0.01 absorbance units, which was produced by solutions containing 1.0 *µ*g/g creatinine. Thus, the LOQ was established as 0.05 *µ*g/g creatinine. The limit of detection (LOD) was 0.5 *µ*g/g creatinine. BIO-RAD Lyphochek Urine Metals Control (Bio-Rad, USA) was prepared from human urine with added mercury. The accuracy of the overall method ranged from 97.1 to 99.9% and the calculated precision was within 5% RSD. The urine samples were analyzed at the certified laboratory at the Faculty of Medicine Technology, Mahidol University.

### 3.6. Statistical Analysis

Descriptive statistics (means and SD) were used to characterize the difference between exposed and unexposed groups including demographic characteristics, mercury vapor levels, and urinary mercury concentrations, frequencies, and percentages.

The data were tested for the normality using a Kolmogorov-Smirnov test. The data were normally distributed. An independent *t*-test was used to compare the means of continuous variables. Pearson's test was used to test the associations between mercury airborne levels and urinary mercury concentrations. Student's *t*-test was used to compare the 2 groups. A *P* value of less than 0.05 was considered statistically significant. Multiple linear regression analysis was used to investigate the effect of independent variables (descriptive characteristics, dietary habit, work characteristics, occupational lifestyle, personal protective equipment (PPE), and personal hygiene) on urinary mercury concentrations. PPE use and personal hygiene practice were characterized as dummy variables (yes/no and always/sometimes) in the model. A *P* value of less than 0.05 was considered statistically significant.

## 4. Results

248 subjects participated in this study. Most of the subjects (51.6%) were aged between 30 and 40 years. The group of exposed subjects consisted of 30 smokers (24.2%) and 94 nonsmoking exposed subjects (80.6%), while the group of unexposed subjects consisted of 78 smokers (62.9%) and 46 nonsmoking unexposed subjects (37.1%).

More unexposed subjects drank alcohol (62.9%) than did the exposed subjects (19.4%). In this study, 57.3% of exposed subjects consumed fish and seafood ≥3 times/month and 42.7% of them consumed fish and seafood ≤3 times/month, while 68.5% of unexposed subjects consumed fish and seafood ≥3 times/month and 31.5% of them consumed fish and seafood 43 times/month (Table 1).

The accuracy of airborne mercury analysis was checked by running 3 samples of Standard Reference Material (SRM). The limit of detection (LOD) was 0.5 *µ*g/L. Recovery varied between 95% and 105%, and the precision was also within acceptable limits of 5% RSD. The occupational exposure standard limits (OELs) for mercury vapor are 25 *µ*g/m^3^ for 8 hrs a day and 40 hrs a week on time/weight average (TWA). 17.3% (*n* = 10/58) of area samplings and 17.7% (*n* = 22/124) of personal air samplings exceeded OELs (Table 2).

The mean urinary mercury levels of the exposed and unexposed subjects were significantly different (*P* < 0.001). All dental health workers had urinary mercury levels less than 20 *µ*g/g of creatinine. The urinary mercury levels were also below the 20 *µ*g/g of creatinine biological exposure indices as recommended by the American Conference of Governmental Industrial Hygienist (ACGIH) [14] (Table 3).

There was no significant difference in urinary mercury levels among job positions (*P* = 0.182). From our observations and walk-through surveys, all dental clinics used air conditioning and electric fans while dental health workers were performing dental practices. Most of dental health workers (65%) had been working for more than 8 hrs per day, and 53% worked for six days per week. Most of them (72.6%) had started their careers before they were 21 years old. Dental health workers who worked for more than 5 years had significantly higher urinary mercury levels than those who had worked 5 years or less (*P* = 0.031). Dental health workers who used a mask or/and gloves had significantly lower urinary mercury levels than those who did not (*P* < 0.001). Dental health workers, who always washed their hands before lunch, had significantly lower urinary mercury levels than those who did only sometimes. Dental health workers who consumed fish and seafood ≥3 times/month had significantly higher urinary mercury levels than those who consumed fish and seafood ≤3 times/month (*P* = 0.022) (Table 4).

To predict the urinary mercury levels of dental health workers, a multiple linear regression model was constructed (Table 5). Significant predictors of urinary mercury levels were duration of work (years), job position, PPE use (mask, gloves, and safety glasses), personal hygiene behavior (snack eating or water drinking and hand washing before lunch and after works), and dietary habit (fish and seafood consumed). Dental health workers who had been working for more than 5 years had significantly higher urinary mercury levels than those had been working for less than 5 years (*P* = 0.011). Dental health workers who used both mask and gloves (*r* = −0.048 and *r* = −0.026, resp.) had significantly lower urinary mercury levels than those who did not (*P* < 0.001). Dental health workers who consumed fish and seafood ≥3 times/month (*r* = 0.0026) had significantly higher urinary mercury levels than those who consumed fish and seafood ≤3 times/month (*P* = 0.013).

**Figure 1:**
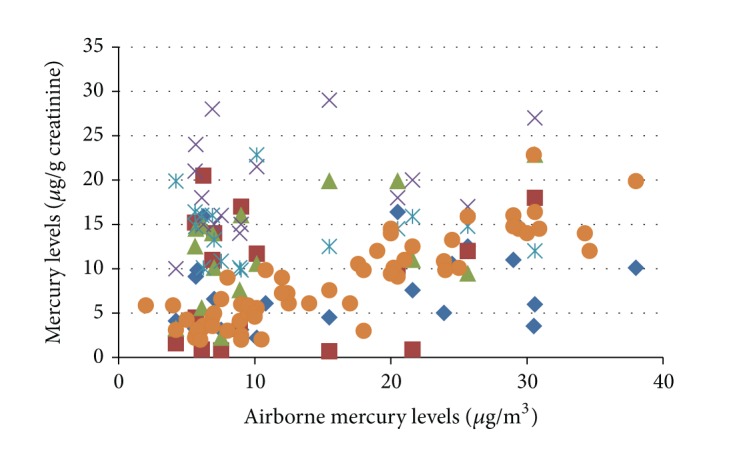
The correlation plot of airborne mercury levels versus dental health workers' mercury levels.

There were significant correlations between levels of mercury in urine and the sampling areas at the mercury storage areas (*r* = 0.499, *P* < 0.049) and personal airborne samplings (*r* = 0.878, *P* < 0.001) (Figure 1).

## 5. Discussion

### 5.1. Area Air Sampling Mercury Levels and Personal Dosimeter Sampling and Urinary Mercury Levels

17.3% of the area air samplings (10/58 samples) exceeded the OELs in dental clinics. The highest mercury concentrations were found at the base of the dental chairs, amalgam storages, and preparation areas where an amalgamator was used. This study had similar findings as the previous study by Langworth, 1997 [17], who reported that the levels of mercury vapor around the base of the chairs may be affected by amalgam places, removing old amalgam restorations and polishing, and floor cleaning. In addition, 17.7% of personal air samples (22/124 samples) exceeded 25 *µ*g/m^3^, the recommended OEL.

In this study, mercury exposure concentrations were determined using a long exposure period to mercury and inorganic mercury method. This study also showed that the urinary mercury levels in dental health workers were higher than in unexposed subjects. The results were similar to the previous study by Zimmer et al., 2002 [18], who reported that body mercury burden of dental health workers was normally higher than in the general population. The mean of urine mercury levels in dental health workers was reported to range from 3 to 22 *µ*g/L compared to 1–5 *µ*g/L for nonoccupational groups.

In this study the urinary mercury levels in dental health workers were 8.24 ± 1.89 *µ*g/g creatinine on average (range 2.0–22.84 *µ*g/g creatinine). Most of them had urinary mercury levels less than 20 *µ*g/g creatinine which is recommended by ACGIH for mercury in urine [14]. Dental health workers who had poor protective practices had a urinary mercury levels up to 22.84 *µ*g/g creatinine. The authors noted that a dental health worker was exposed to mercury during the preparation of the dental amalgam, the insertion and removal of amalgam restoration, and storage of mercury. Mercury exposure was directly related to the hygienic practices. This dental health worker was exposed to up to 22.84 *µ*g/g creatinine of mercury for more than 8 hours/day, 6 days/week for 15 years. The dental health worker normally had poor personal hygiene practice and was therefore the highest exposed dental health workers of the group. The present study agrees with Fung and Molvar, 1992 [19], who reported that good hygiene is essential in minimizing exposure to mercury vapour.

Saengsirinavin and Pringsulaka, 1988 [20], conducted a study in Thailand that showed that mercury accumulation in urine and hair of dental health workers was the highest amongst the dental assistants group (means = 17.1 ± 2.44 *µ*g/L). The mean urine mercury levels found in dentists, dental students, and dental technicians were 10.1 ± 1.42, 11.1 ± 1.69, and 3.2 ± 0.69 *µ*g/L, respectively.

### 5.2. Factors Associated with Mercury in Urine

Dental health workers who consumed fish and seafood ≥3 times/month had significantly higher urinary mercury levels than those who consumed fish and seafood ≤3 times/month, similar to the study conducted by Zolfaghari et al., 2007 [21], who reported that fish consumption and number of patients visited per day had a significant effect on hair (*P* = 0.02 and *P* = 0.02, resp.) and nails (*P* = 0.03 and *P* = 0.02, resp.) mercury levels. In this study, there was no significant difference in urinary mercury levels among the subjects who smoked and nonsmokers similar to the study by Zolfaghari et al., 2007 [21], who reported that the mercury levels among Iranian dentists were not affected by smoking.

For the duration of work, workers who had worked ≥5 years had significantly higher urinary mercury levels than those who had worked <5 years. This may be due to a lack of appropriate PPE use and environmental area prevention, leading to higher accumulations in their bodies [22]. The use of PPE at work can help prevent contamination. Dental health workers who used masks and gloves had significantly lower urinary mercury levels than those who did not. The present study agrees with Rogers, 1983 [23], who reported that the risk of exposure to hazardous materials will decrease if the appropriate behaviors are adopted and practiced. However, the types of PPE in use in these dental clinics were inappropriate for field work. Mercury can accumulate on the surfaces of PPE used by the dental health workers. In addition, mercury may penetrate a cotton mask and enter a worker's airway. Dental health workers using these inappropriate protective devices may also mistakenly believe that they are protected.

Personal hygiene and behavioral risk factors were also associated with urinary mercury levels (Table 5). Dental health workers who washed their hands before lunch had significantly lower urinary mercury levels than those who did sometimes, similar to the study conducted by Eley [24], who reported that good personal hygiene was the essential factor in minimizing exposure to mercury airborne vapors.

These poor protective practices meant that dental health workers were likely to carry mercury contamination elsewhere, potentially exposing their homes and families. Paraoccupational or take-home exposure among workers' families may cause mercury poisoning among family members [25–27].

There were significant correlations between urinary mercury levels and environmental mercury levels. Nixon et al. [28] reported that increased ventilation will reduce the amount of airborne mercury vapor in the environment. There were significant correlations between urinary mercury levels and the environmental samplings that were conducted at the mercury storage areas and personal vapor samplings similar to the study by Ritchie et al. [29] who reported the associations between urinary mercury and environmental mercury in the amalgam storage and preparation areas, surgery air, and personal dosimeter readings. This was also supported by Tsuji et al. [30], who reported that ten studies reporting paired air and urine mercury data (149 samples total) met criteria for data quality and sufficiency. The log-transformed data set showed a strong correlation between mercury in air and in urine (*r* = 0.774), although the relationship was best fit by a series of parallel lines with different intercepts for each study (*R*_2_ = 0.807). However, since most dental chair-side personnel do not touch dental amalgam during mixing and placement, it is considered that the main sources of mercury exposure are aerosols, created in the immediate working environment during and in particular the removal of restorations of dental amalgam, and the exhaust air from dental vacuum systems. These mercury vapor releases can be substantial and well in excess of human exposure limits [31].

In addition, aerosols and exhaust air from dental vacuum systems will be inhaled despite wearing face masks, which may provide little, if any, barrier to mercury vapors entering the lungs and being absorbed. However, several previous studies have indicated that good personal hygiene was an essential factor in minimizing exposure to mercury vapor [32–34].

## 6. Conclusions

This study demonstrated that urinary mercury levels were associated with airborne mercury levels and hygiene behaviors of dental health workers. This study showed that improving dental health workers hygiene habits can reduce urinary mercury levels. This study recommends conducting education and training about personal hygiene to minimize occupational mercury vapor exposure. In addition, engineering controls are also recommended to reduce mercury vapor exposure.

Further study increasing the sample size of participants would also be beneficial for a better understanding of this health risk.

## Figures and Tables

**Table 1:** Descriptive characteristics of the exposed and unexposed subjects (*n* = 248).

Parameters	Exposed (*n* = 124)	Unexposed (*n* = 124)
Sex		
Male	30 (24.2)	30 (24.2)
Female	94 (75.8)	94 (75.8)
Age (yrs)		
20–30	40 (32.3)	20 (16.1)
>30–40	66 (53.2)	62 (50.0)
>40–50	16 (12.9)	42 (33.9)
>50	2 (1.6)	0
Cigarette smoking		
No	94 (75.8)	46 (37.1)
Yes	30 (24.2)	78 (62.9)
Alcohol drinking		
No	100 (80.6)	46 (37.1)
Yes	24 (19.4)	78 (62.9)
Dietary habit (fish or seafood consumption)		
≤3 times/month	53 (42.7)	39 (31.5)
≥3 times/month	71 (57.3)	85 (68.5)

**Table 2:** Environmental mercury vapor samplings and percentage of mercury airborne levels exceeded (% OELs).

Personal and area samplings		*µ*g/m^3^ TWA				
	*n*	Mean	Median	Min	Max	Number of mercury airborne levels exceeded (% OELs)
Chairs	24	9.42	5.70	0.20	31.10	2 (8.3)
Amalgam storages	17	19.28	18.00	10.00	29.00	6 (35.3)
Preparation areas	17	8.88	10.50	0.70	20.50	2 (11.8)
Total areas samplings	**58**	**8.58**	**6.40**	**0.20**	**29.00**	**10/58 (17.3)**
Personal air samplings	124	15.60	12.20	2.00	38.00	22/124 (17.7)

**Table 3:** Urinary mercury levels of exposed and unexposed subjects.

Metal	Exposed (*n* = 124)	Unexposed (*n* = 124)	*P* value
Mercury (*µ*g/g creatinine)			
Mean	8.24	2.00	<0.001*
Standard deviation	1.89	0.11	
Range	2.00–22.84	1.00–10.00	

*Significant at *P* value of <0.05.

**Table 4:** Descriptive characteristics of urinary mercury levels, PPEs used, and personal hygiene, behaviors, and dietary habit.

Parameter	Number of mercury exposed dental health workers	Urinary mercury mean (*µ*g/g creatinine)	SD	*P* value
Position				
Dentists	16	5.37	1.29	0.182
Dental hygienists	70	8.75	1.95	
Dental assistants	38	8.66	1.16	
Duration of work (yrs)				
≤5	10	3.15	0.02	0.031*
>5	114	8.47	1.09	
PPEs uses				
Mask				
Yes	110	7.19	0.89	<0.001*
No	14	16.84	1.28	
Glove				
Yes	30	6.91	1.25	<0.001*
No	94	12.59	1.32	
Safety glasses				
Yes	10	7.64	1.68	0.223
No	114	5.30	0.87	
Ate snacks/drank water during work				
Sometimes	88	7.80	1.76	0.252
Always	36	9.46	1.17	
Wash hands before lunch				
Sometimes	62	9.58	0.97	0.036*
Always	62	6.98	1.53	
Wash hands before dinner				
Sometimes	56	9.26	1.51	0.166
Always	68	7.48	1.25	
Clean cloths				
Everyday	24	7.64	0.68	0.134
2-3 days	16	5.30	0.87	
Week or more	84	9.04	0.08	
Dietary habit				
≤3 times/month	53	5.23	1.68	0.022*
≥3 times/month	71	9.85	0.88	

*Significant at *P* value < 0.05.

**Table 5:** Multiple linear regression of dietary habit, occupational life style, PPEs used, and personal hygiene behaviors on urinary mercury levels in dental health personnel.

Parameters	Regression coefficient	SE	*P* value
Position (dentists, dental hygienist, and dental assistants)	0.0005	0.0002	0.082
Duration of work (more than 5 yrs versus less than 5 yrs)	0.0024	0.0010	0.011*
Mask using (yes versus no)	−0.0477	0.0118	<0.001*
Glove using (yes versus no)	−0.0259	0.0193	<0.001*
Snack eating/water drinking at work (always versus sometimes)	0.1470	0.0294	0.054
Hand washing before lunch (always versus sometimes)	−0.0483	0.0114	<0.001*
Hand washing after work (always versus sometimes)	−0.0479	0.0159	0.0001*
Dietary habit (fish and seafood consumption; ≤3 times/month versus ≥3 times/month)	0.0026	0.0015	0.013*

*Significant at *P* value of <0.05.
